# Epistatic SNP interaction of *ERCC6* with *ERCC8* and their joint protein expression contribute to gastric cancer/atrophic gastritis risk

**DOI:** 10.18632/oncotarget.17814

**Published:** 2017-05-11

**Authors:** Jing-Jing Jing, You-Zhu Lu, Li-Ping Sun, Jing-Wei Liu, Yue-Hua Gong, Qian Xu, Nan-Nan Dong, Yuan Yuan

**Affiliations:** ^1^ Tumor Etiology and Screening Department of Cancer Institute and General Surgery, The First Affiliated Hospital of China Medical University, Key Laboratory of Cancer Etiology and Prevention, China Medical University, Liaoning Provincial Education Department, Shenyang, Liaoning, China

**Keywords:** gastric cancer, ERCC6, ERCC8, SNP, expression

## Abstract

Excision repair cross-complementing group 6 and 8 (ERCC6 and ERCC8) are two indispensable genes for the initiation of transcription-coupled nucleotide excision repair pathway. This study aimed to evaluate the interactions between single nucleotide polymorphisms of *ERCC6* (rs1917799) and *ERCC8* (rs158572 and rs158916) in gastric cancer and its precancerous diseases. Besides, protein level analysis were performed to compare ERCC6 and ERCC8 expression in different stages of gastric diseases, and to correlate SNPs jointly with gene expression. Sequenom MassARRAY platform method was used to detect polymorphisms of *ERCC6* and *ERCC8* in 1916 subjects. *In situ* ERCC6 and ERCC8 protein expression were detected by immunohistochemistry in 109 chronic superficial gastritis, 109 chronic atrophic gastritis and 109 gastric cancer cases. Our results demonstrated pairwise epistatic interactions between *ERCC6* and *ERCC8* SNPs that *ERCC6* rs1917799-*ERCC8* rs158572 combination was associated with decreased risk of chronic atrophic gastritis and increased risk of gastric cancer. *ERCC6* rs1917799 also showed a significant interaction with *ERCC8* rs158916 to reduce gastric cancer risk. The expressions of ERCC6, ERCC8 and ERCC6-ERCC8 combination have similarities that higher positivity was observed in chronic superficial gastritis compared with chronic atrophic gastritis and gastric cancer. As for the effects of *ERCC6* and *ERCC8* SNPs on the protein expression, single SNP had no correlation with corresponding gene expression, whereas the *ERCC6* rs1917799–*ERCC8* rs158572 pair had significant influence on ERCC6 and ERCC6-ERCC8 expression. In conclusion, *ERCC6* rs1917799, *ERCC8* rs158572 and rs158916 demonstrated pairwise epistatic interactions to associate with chronic atrophic gastritis and gastric cancer risk. The *ERCC6* rs1917799–*ERCC8* rs158572 pair significantly influence ERCC6 and ERCC6-ERCC8 expression.

## INTRODUCTION

Nucleotide excision repair (NER) is a critical and versatile system that monitors and repairs a broad spectrum of DNA damage. NER is composed of global genome nucleotide excision repair (GGR) and transcription-coupled nucleotide excision repair (TCR) [1]. TCR specifically repairs RNA polymerase II (RNAPII) blocking DNA lesions and ensures the transcribed strand of the active gene to be repaired preferentially than the other sites of the genome [2]. *ERCC6* (Excision repair cross-complementing group6, alternatively known as *CSB*) and *ERCC8* (Excision repair cross-complementing group8, alternatively known as *CSA*) gene, are two indispensable core genes for the initiation of TCR pathway [3]. They were first described in the Cockayne syndrome (CS), a human autosomal recessive disease. ERCC6 and ERCC8 proteins, with direct interaction, jointly participate in DNA repair, transcriptional regulation, maintenance of the chromosome stability and chromatin remodeling [4–6].

Sequence variations and differential expression of the genes accounting for major components of human DNA repair system may be implicated in the carcinogenesis and development of cancer [7, 8]. In our previous study, we systematically analyzed the association of 43 SNPs of ten key NER pathway genes including *ERCC6*, *ERCC8* with survival of gastric cancer (GC) patients [9], and found that *ERCC6* SNP could predict GC prognosis. Moreover, *ERCC6* rs1917799 and *ERCC8* rs158572 polymorphisms were associated with increased GC risk separately [10, 11]. However, it is unclear whether there are interactions among *ERCC6* and *ERCC8* SNPs, by which it may enhance the risk warning for GC or its precancerous diseases because SNP-SNP interactions could be more valuable than a single SNP in cancer prediction [12–14]. In addition, it is also elusive whether the SNP-SNP combination is functional to influence ERCC6 and ERCC8 expression and whether the protein expression individually or jointly is associated with the development of GC, by which it may provide a genetic clue for phenotypic variations of gastric diseases and gastric carcinogenesis.

In this case-control study based on Chinese populations, we evaluated the two- and three-dimensional interactions between SNPs of *ERCC6* and *ERCC8* in GC and its precancerous diseases. We also performed protein level analysis to compare ERCC6 and ERCC8 expression (alone or in combination) in chronic superficial gastritis (CSG), chronic atrophic gastritis (CAG) and GC, and to correlate SNPs jointly with gene expression. We hoped to find potential combinations of biomarkers that could provide experimental evidence for the early diagnosis of GC.

## RESULTS

### Interaction analyses of *ERCC6* and *ERCC8* SNPs in different gastric diseases

#### Interactions between *ERCC6* and *ERCC8* SNPs in the risk of GC and CAG

We firstly focused on the pairwise interaction effects for SNPs of *ERCC6* and *ERCC8*. In the two-way interaction analyses, significant interaction was observed. The results indicated that *ERCC6* rs1917799 and *ERCC8* rs158572 polymorphisms had interaction effects for CAG and GC (*P_interaction_*=0.013 and 0.021, separately). In addition, *ERCC6* rs1917799 showed a significant interaction with *ERCC8* rs158916 in relation to GC risk (*P_interaction_*=0.042) (Table 1). These results demonstrated that combination of the SNPs could have a synergistic effect on gastric carcinogenesis.

**Table 1:** Two-way interactions between *ERCC6* and *ERCC8* polymorphisms in the risk of GC and CAG

Gene	Genotypes	Number of Participants	ERCC6 rs1917799			
			TT	GT/GG	TT/GT	GG
**CAG vs CSG (n=700 vs.749)**						
**ERCC8 rs158572**	**AA**	**No. of controls/cases**	229/228	401/382	524/510	106/100
		**OR (95% CI)**	1(Ref)	0.92(0.72-1.19)	1(Ref)	0.96(0.70-1.33)
	**GA/GG**	**No. of controls/cases**	31/37	88/53	97/73	22/17
		**OR (95% CI)**	1.42(0.81-2.5)	0.54(0.36-0.83)	0.78(0.54-1.12)	0.79(0.39-1.60)
			***P_interaction_*=0.013**		*P_interaction_=*0.899	
			**interaction index=0.41**		interaction index=1.06	
	**AA/GA**	**No. of controls/cases**	259/263	486/431	618/578	127/116
		**OR (95% CI)**	1(Ref)	0.82(0.65-1.04)	1(Ref)	1.71(0.36-8.21)
	**GG**	**No. of controls/cases**	1/2	3/4	3/5	1/1
		**OR (95% CI)**	2.29(0.18-29.2)	1.07(0.20-5.67)	0.97(0.72-1.31)	1.00(0.05-20.4)
			*P_interaction_*=0.719		*P_interaction_*=0.772	
			interaction index=0.57		interaction index=0.60	
**ERCC8 rs158916**	**TT**	**No. of controls/cases**	202/203	373/348	476/457	99/94
		**OR (95% CI)**	1(Ref)	0.87(0.69-1.14)	1(Ref)	1.02(0.73-1.43)
	**CT/CC**	**No. of controls/cases**	58/62	116/87	145/126	29/23
		**OR (95% CI)**	1.00(0.64-1.56)	1.07(0.20-5.67)	0.87(0.65-1.17)	0.68(0.37-1.25)
			*P_interaction_*=0.306		*P_interaction_*=0.475	
			interaction index=0.75		interaction index=0.77	
	**TT/CT**	**No. of controls/cases**	254/261	479/425	607/571	126/115
		**OR (95% CI)**	1(Ref)	0.81(0.64-1.05)	1(Ref)	0.97(0.72-1.31)
	**CC**	**No. of controls/cases**	6/4	10/10	14/12	2/2
		**OR (95% CI)**	0.44(0.11-1.81)	0.73(0.28-1.91)	0.74(0.32-1.74)	0.54(0.07-4.23)
			*P_interaction_*=0.406		*P_interaction_*=0.794	
			interaction index=2.06		interaction index=0.74	
**GC vs CSG (n= 467vs.749)**						
**ERCC8 rs158572**	**AA**	**No. of controls/cases**	229/119	401/268	524/315	106/72
		**OR (95% CI)**	1(Ref)	1.27(0.94-1.71)	1(Ref)	1.20(0.83-1.73)
	**GA/GG**	**No. of controls/cases**	31/28	88/52	97/65	22/15
		**OR (95% CI)**	2.20(1.19-4.1)	1.16(0.74-1.83)	1.28(0.87-1.88)	1.11(0.53-2.36)
			***P_interaction_*=0.021**		*P_interaction_*=0.482	
			**interaction index=0.41**		interaction index=0.73	
	**AA/GA**	**No. of controls/cases**	259/145	486/315	618/373	127/87
		**OR (95% CI)**	1(Ref)	1.11(0.84-1.45)	NA	NA
	**GG**	**No. of controls/cases**	1/2	3/5	3/7	1/0
		**OR (95% CI)**	1.50(0.13-17.8)	3.45(0.69-17.22)	NA	NA
			*P_interaction_*=0.625		NA	
			interaction index=2.09		NA	
**ERCC8 rs158916**	**TT**	**No. of controls/cases**	202/104	373/261	476/299	99/66
		**OR (95% CI)**	1(Ref)	1.30(0.95-1.78)	1(Ref)	1.12(0.76-1.64)
	**CT/CC**	**No. of controls/cases**	58/43	116/59	145/174	29/21
		**OR (95% CI)**	1.27(0.77-2.10)	0.86(0.56-1.32)	0.82(0.59-1.15)	1.00(0.53-1.90)
			***P_interaction_*=0.042**		*P_interaction_*=0.816	
			**interaction index=0.52**		interaction index=1.10	
	**TT/CT**	**No. of controls/cases**	254/142	479/313	607/371	126/84
		**OR (95% CI)**	1(Ref)	1.12(0.85-1.47)	1(Ref)	1.11(0.79-1.56)
	**CC**	**No. of controls/cases**	6/5	10/7	14/16	2/3
		**OR (95% CI)**	1.10(0.29-4.11)	1.11(0.37-3.30)	0.86(0.34-2.17)	2.76(0.35-21.8)
			*P_interaction_*=0.905		*P_interaction_*=0.363	
			interaction index=0.90		interaction index=2.89	

CSG: chronic superficial gastritis; CAG: chronic atrophic gastritis; GC: gastric cancer.

NA not available.

Indeed, such an interaction effect between polymorphisms of two or more genes may be indicative of epistasis [15], which refers to an interaction between a pair of loci, in which the phenotypic effect of one locus depends on the genotype at the second locus. As such, the genetic effects of the polymorphisms on disease risks would have been missed had they not been tested jointly. We therefore examined the epistatic effects between pairs of interacting factors. For *ERCC6* rs1917799 and *ERCC8* rs158572, GT/GG genotypes of rs1917799 and GA/GG genotypes of rs158572 each conferred a reduced risk of CAG (OR = 0.35 and 0.59, respectively), but only if they were both present; rs158572 GA/GG genotypes were associated with an increased risk of GC, but only in the presence of TT genotypes of rs1917799. Regarding *ERCC8* rs158916, the CT/CC genotype was associated with a reduced risk of CAG (OR = 0.66), but only in the presence of the *ERCC6* rs1917799 GT/GG genotype (Table 2).

**Table 2:** Epistatic effect of pair-wise interacting factors on the risks of GC and CAG

Interacted pair-wise SNPs	Comparison	Subset	CAG vs. CSG		GC vs. CSG	
			*P*	OR(95%CI)	*P*	OR(95%CI)
***ERCC6* rs1917799 interacted with *ERCC8* rs158572**	***ERCC6* rs1917799 GT/GG vs. TT**	***ERCC8* rs158572 AA**	0.526	0.92(0.72-1.09)	0.111	1.27(0.95-1.72)
		***ERCC8* rs158572 GA/GG**	**0.003**	**0.35(0.18-0.69)**	0.068	0.52(0.26-1.05)
	***ERCC8* rs158572 GA/GG vs. AA**	***ERCC6* rs1917799 TT**	0.240	1.40(0.80-2.45)	**0.014**	**2.20(1.18-4.11)**
		***ERCC6* rs1917799 GT/GG**	**0.011**	**0.59(0.39-0.88)**	0.694	0.92(0.60-1.40)
***ERCC6* rs1917799 interacted with *ERCC8* rs158916**	***ERCC6* rs1917799 GT/GG vs. TT**	***ERCC8* rs158916TT**	0.310	0.87(0.67-1.14)	0.098	1.30(0.95-1.78)
		***ERCC8* rs158916 CT/CC**	0.093	0.65(0.40-1.07)	0.153	0.67(0.38-1.16)
	***ERCC8* rs158916 CT/CC vs. TT**	***ERCC6* rs1917799 TT**	0.973	1.01(0.65-1.57)	0.334	1.28(0.77-2.13)
		***ERCC6* rs1917799 GT/GG**	0.088	0.74(0.53-1.05)	**0.035**	**0.66(0.45-0.97)**

CSG: chronic superficial gastritis; CAG: chronic atrophic gastritis; GC: gastric cancer.

Moreover, interactions involving multiple polymorphisms were also explored among the three SNPs of *ERCC6* and *ERCC8* involved in pairwise interactions. However, *ERCC6* rs1917799-ERCC8 rs158572-*ERCC8* rs158916 had no obvious interaction in relation to CAG or GC risk (both *P_interaction_*>0.05) (Table 3). All these evidences suggest that the SNP-SNP interaction is mainly in an epistatic pairwise pattern.

**Table 3:** The three dimensions interactions of the *ERCC6* rs1917799-*ERCC8* rs158572-*ERCC8* rs158916 with the risk of GC and CAG

SNP genotypes			CAG vs. CSG		GC vs. CSG	
			*P*	OR(95%CI)	*P*	OR(95%CI)
***ERCC6* rs1917799-*ERCC8* rs158572-*ERCC8* rs158916**						
**TT**	**AA**	**TT**		1(ref)		1(ref)
**TT**	**AA**	**CT/CC**	0.375	1.24(0.77-1.97)	0.188	1.44(0.84-2.49)
**TT**	**GA/GG**	**TT**	0.031	1.97(1.07-3.63)	0.006	2.75(1.33-5.55)
**TT**	**GA/GG**	**CT/CC**	0.088	0.23(0.04-1.25)	0.362	1.74(0.53-5.72)
**GT/GG**	**AA**	**TT**	0.718	1.06(0.79-1.41)	0.013	1.56(1.10-2.20)
**GT/GG**	**AA**	**CT/CC**	0.153	0.75(0.51-1.11)	0.962	1.01(0.64-1.59)
**GT/GG**	**GA/GG**	**TT**	0.032	0.61(0.39-0.96)	0.252	1.33(0.81-2.20)
**GT/GG**	**GA/GG**	**CT/CC**	0.074	0.27(0.07-1.14)	0.818	0.86(0.24-3.10)
			*P_interaction_*=0.119		*P_interaction_*=0.429	
			interaction index=6.61		interaction index=2.23	

CSG: chronic superficial gastritis; CAG: chronic atrophic gastritis; GC: gastric cancer.

#### Effect modification of *H. pylori* infection on the SNP–SNP interactions

*Helicobacter pylori* (*H. pylori*) infection is the most important environmental factor which may interact with hereditary factors to increase host susceptibility. We next investigated three-way interaction of *ERCC6* and *ERCC8* SNPs with *H. pylori* infection. There was no three-way combination had obvious interaction with CAG or GC risk (all *P_interaction_*>0.05) (Supplementary Table 2).

### Individual and joint expression of ERCC6 and ERCC8 in different gastric diseases

#### Individual expression of ERCC6 and ERCC8 in different gastric tissues

**Figure 1:**
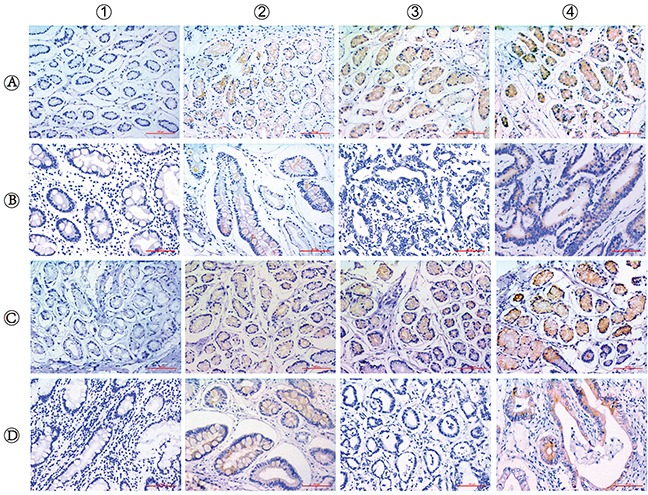
Immunohistochemical staining for ERCC6 and ERCC8 expression in CSG, CAG and GC The staining of ERCC6 and ERCC8 are mainly located in the cytomembrane and cytoplasm. (A1-4) CSG with negative (−), weakly positive (+), moderately positive (++) and strongly positive (+++) ERCC6 expression; (B1-2) CAG with negative and positive ERCC6 expression; (B3-4) GC with negative and positive ERCC6 expression; (C1-4) CSG with negative (−), weakly positive (+), moderately positive (++) and strongly positive (+++) ERCC8 expression; (D1-2) CAG with negative and positive ERCC8 expression; (D3-4) GC with negative and positive ERCC8 expression. (Magnification, ×200; bar = 100μm).

**Figure 2:**
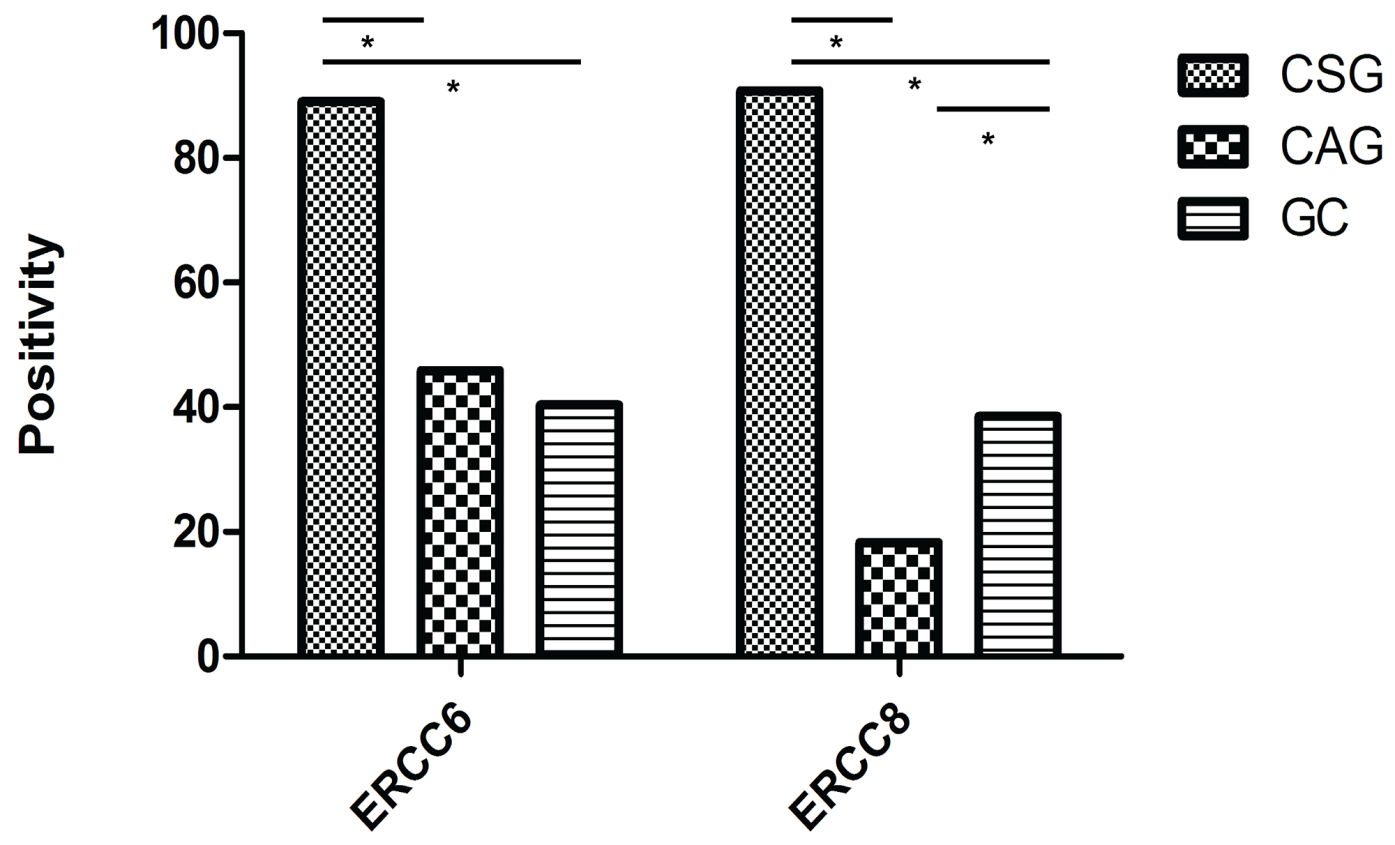
Positivity for ERCC6 and ERCC8 expression in CSG, CAG and GC (*, *P*<0.05).

We detected ERCC6 and ERCC8 *in situ* expression in CSG, precancerous CAG and GC by immunohistochemistry separately. ERCC6 and ERCC8 immunostaining were predominantly observed in the cytoplasm (Figure 1). Our results showed that expression of ERCC6 in CSG was higher than that in CAG and GC (*P*<0.001), but there was no obvious difference between CAG and GC (*P*=0.412). The expression of ERCC8 in CSG was higher than that in CAG and GC (*P*<0.001), but was lower in CAG than in GC (*P*=0.001) (Table 4) (Figure 2). GC cases were further classified into CAG group/non-CAG group by histopathological examination of adjacent non-cancerous tissues, and intestinal/diffuse group by Lauren's Classification. In the stratified analysis, we found that positivity of ERCC6 and ERCC8 were higher in CSG than in different type of GC (all *P*<0.001). ERCC6 positivity was lower in diffuse GC than that in CAG (*P*=0.006), and ERCC8 positivity was lower in CAG compared with CAG-GC (*P*<0.001), intestinal-GC (*P*<0.001) and diffuse-GC (*P*=0.024). ERCC6 and ERCC8 expressions were significantly higher in intestinal GC than in diffuse GC (both *P*<0.05) (Table 4).

**Table 4:** Individual and joint expression of ERCC6 and ERCC8 in different gastric diseases

Group	case	(−)	(+)	(+ +)	(+ + +)	Positive (%)	Negative (%)		*P*	
**ERCC6 expression**										
**CSG**	109	12	52	31	14	97(89.0)	12(11.0)	ref.		
**CAG**	109	59	40	10	0	50(45.9)	59(54.1)	**<0.001**	ref.	
**GC**	109	65	40	4	0	44(40.4)	65(59.6)	**<0.001**	0.412	
**CAG-GC**	68	39	27	2	0	29(42.6)	39(57.4)	**<0.001**	0.756	ref.
**non CAG-GC**	41	26	13	2	0	15(36.6)	26(63.4)	**<0.001**	0.306	0.532
**intestinal-GC**	24	10	14	0	0	14(58.3)	10(41.7)	**<0.001**	0.269	ref.
**diffuse-GC**	83	61	18	4	0	22(26.5)	61(73.5)	**<0.001**	**0.006**	**0.004**
**ERCC8 expression**										
**CSG**	109	10	45	37	17	99(90.8)	10(9.2)	ref.		
**CAG**	109	86	20	0	0	20(18.3)	89(81.7)	**<0.001**	ref.	
**GC**	109	67	40	1	1	42(38.5)	67(61.5)	**<0.001**	**0.001**	
**CAG-GC**	68	38	29	1	0	30(44.1)	38(55.9)	**<0.001**	**<0.001**	ref.
**non CAG-GC**	41	29	11	0	1	12(29.3)	29(70.7)	**<0.001**	0.146	0.123
**intestinal-GC**	24	9	15	0	0	15(62.5)	9(37.5)	**<0.001**	**<0.001**	ref.
**diffuse-GC**	83	56	25	1	1	27(32.5)	56(67.5)	**<0.001**	**0.024**	**0.008**
**ERCC6-ERCC8 expression**										
**CSG**	109					91(83.5)*	18(16.5)**	ref.		
**CAG**	109					14(12.8)*	95(87.2)**	**<0.001**	ref.	
**GC**	109					21(19.3)*	88(80.7)**	**<0.001**	0.197	
**CAG-GC**	68					15(22.1)*	53(77.9)**	**<0.001**	0.107	ref.
**non CAG-GC**	41					6(14.6)*	35(85.4)**	**<0.001**	0.774	0.341
**intestinal-GC**	24					8(33.3)*	16(66.7)**	**<0.001**	**0.014**	ref.
**diffuse-GC**	83					10(12.0)*	73(88.0)**	**<0.001**	0.869	**0.014**

CSG: chronic superficial gastritis; CAG: chronic atrophic gastritis; GC: gastric cancer.* double positive for ERCC6 and ERCC8 expression; ** single or double negative for ERCC6 and ERCC8 expression.

#### Joint expression of ERCC6 and ERCC8 in different gastric tissues

For the joint expression of ERCC6 and ERCC8, the results showed that ERCC6-ERCC8 double positive expression in CSG was significantly higher than in CAG and GC (both *P*<0.001), while no obvious difference was observed between CAG and GC (*P*=0.197). For subgroup analysis, ERCC6-ERCC8 expression was higher in CSG compared with different type of GC (all *P*<0.001), higher in intestinal-GC than in CAG (P=0.014) and diffuse-GC (P=0.014) (Table 4).

### Effects of ERCC6 and ERCC8 SNPs on the protein expression

#### Effects of a single SNP on ERCC6 and ERCC8 expression

To examine whether the gene expression are correlated with *ERCC6* and *ERCC8* SNPs, 109 CSG subjects with both SNP genotype information and relatively high protein expression were used for analysis, which can rule out the interference of disease's factor. In separate analysis of a single SNP, no correlation between the three SNPs and corresponding gene expression was observed (all *P*>0.05) (Supplementary Table 1).

#### Joint effects of SNP combination on ERCC6 and ERCC8 expression

As for joint analysis of two SNPs, in the CSG group, the *ERCC6* rs1917799–*ERCC8* rs158572 pair had significant effect on ERCC6 and ERCC6-ERCC8 expression (*P*=0.021 and 0.011, respectively). The lowest protein expression was observed in TT–GA/GG combined genotype, and GT/GG–GA/GG genotype had the highest protein expression (Table 5).

**Table 5:** Joint effects of SNP combination on ERCC6 and ERCC8 expression

SNP genotypes		Total	(−)	(+)	(+ +)	(+ + +)	ERCC6 positive n(%)	(−)	(+)	(+ +)	(+ + +)	ERCC8 positive n(%)	ERCC6-ERCC8 positive* n(%)
***ERCC6*rs1917799-*ERCC8* rs158572**													
**TT**	**AA**	46	6	23	10	7	40(87.0)	4	19	14	9	42(91.3)	38(82.6)
**TT**	**GA/GG**	7	3	1	3	0	4(57.1)	2	3	2	0	5(74.1)	3(42.9)
**GT/GG**	**AA**	44	3	24	13	4	41(93.2)	4	19	14	7	40(90.9)	38(86.4)
**GT/GG**	**GA/GG**	12	0	4	5	3	12(100.0)	0	4	7	1	12(100.0)	12(100.0)
							***P*=0.021**					*P*=0.223	***P*=0.011**
***ERCC6*rs1917799-*ERCC8* rs158916**													
**TT**	**TT**	41	5	22	9	5	36(87.8)	4	20	10	7	37(92.0)	33(80.5)
**TT**	**CT/CC**	0	\	\	\	\	\	\	\	\	\	\	\
**GT/GG**	**TT**	53	7	22	17	7	46(86.8)	5	18	25	5	48(90.6)	44(83.0)
**GT/GG**	**CT/CC**	15	0	8	5	2	15(100.0)	1	7	2	5	14(93.3)	14(93.3)
							*P*=0.337					*P*=0.935	*P*=0.514

*double positive for ERCC6 and ERCC8 expression

## DISCUSSION

As important members of the NER repair pathway, ERCC6 and ERCC8 are the only two necessary factors for TCR recognition. The novelty of the present study is that the results of genetic model dependent-analyses at protein levels provide a new prospective for a link between interaction of *ERCC6*-*ERCC8* SNPs, interaction of *ERCC6*-*ERCC8* gene expression and phenotypic variations of gastric diseases. To the best of our knowledge, this is the first study to investigate the relationship between *ERCC6*-*ERCC8* SNP interactions, joint protein expression and GC/CAG risk. To some extent, it may help us to gain an in-depth understanding towards the association between *ERCC6* and *ERCC8* functional genotypes, coding protein and gastric carcinogenesis.

Evidence is accumulating that *ERCC6* and *ERCC8* polymorphisms have a close relationship with the susceptibility, progression and prognosis of various malignancies [16–20]. In our previous pilot study, three potential functional SNPs, *ERCC6* rs1917799, *ERCC8* rs158572 and rs158916 were detected and two of them were supposed to be associated with GC risk [10, 11]. In the current study, we further analyzed the interaction effect of the SNPs, and found that *ERCC6* rs1917799 and *ERCC8* rs158572 polymorphisms were associated with CAG and GC. *ERCC6* rs1917799 also showed a significant interaction with *ERCC8* rs158916 in relation to GC risk. It is worth noting that such an interaction effect between polymorphisms of two or more genes may be indicative of epistasis [15], which has been involved in susceptibility to various malignancies [21, 22]. In this study, the strongest epistatic interaction was the pairwise *ERCC6* rs1917799-*ERCC8* rs158572 combination. Both the rs1917799 GT/GG genotype and the rs158572 GA/GG genotype were associated with a reduced risk of CAG, but only in combination. Another two pairs of factors with epistatic effects were identified: rs158572 GA/GG genotypes were associated with an increased risk of GC, but only in the presence of TT genotypes of rs1917799; and rs158916 CT/CC genotype was associated with a reduced risk of CAG, but only in the presence of the rs1917799 GT/GG genotype. We also performed interaction analysis involving multiple polymorphisms of *ERCC6* and *ERCC8* and the effect modification of *H. pylori* infection. However, no significant interaction was observed for the three SNPs or SNP-SNP-*H. pylori* combination. These results demonstrated that the three polymorphisms contribute to gastric carcinogenesis mainly in an epistatic pairwise pattern. In a word, these evidences suggest that the combination of *ERCC6* and *ERCC8* SNPs could have a synergistic effect on gastric carcinogenesis.

Furthermore, individual and joint expression of ERCC6 and ERCC8 were also evaluated by immunohistochemistry in different gastric diseases, which have never been reported before. Our results showed the expressions of ERCC6, ERCC8 and ERCC6-ERCC8 combination have similarities, that higher positivity was observed in CSG compared with CAG and GC. Specifically, ERCC6 positivity decreased as CSG→CAG→GC development, whereas lowest positivity of ERCC8 was observed in CAG group. For ERCC6-ERCC8 joint expression, both CAG and GC group had very low positivity. Therefore, we supposed that ERCC6-ERCC8 joint expression was more suitable for discriminating normal and diseased mucosa, and ERCC8 was a better marker to distinguish CAG from other gastric diseases. Although ERCC6 and ERCC8 might play important roles in the initiation of GC and could serve as biomarkers for this disease, the underlying mechanisms remain unexplored. Physiologically, the expression of DNA repair genes reflects the cellular ability to meet repair demand once cells are stimulated by carcinogens. ERCC6 and ERCC8 defects specifically disrupt the coupling of transcription and repair, resulting in a loss of normal rapid repair [23]. Recently, it was reported that ERCC6 and ERCC8 deficient mice were more susceptible to both UV- and chemically-induced skin cancer [24, 25]. Javeri et al. [26] found that *ERCC6* knockdown in human keratinocytes decreased DNA repair capacity for UV-induced cyclobutane dimers as well as 8-oxo-deoxyguanine, providing mechanistic evidence of a role for ERCC6 in skin carcinogenesis. In addition, different histological type of gastric cancer is thought to differ in pathogenesis, based on dissimilar variants and expression of various genes. A clear difference in genomic instability and DNA mismatch repair between intestinal-GC and diffuse-GC was found [27]. We observed that ERCC6 and ERCC8 positive rates were higher in intestinal-GC than diffuse-GC, from a side confirmed that intestinal subtype is more associated with genomic instability and DNA repair. In brief, we hypothesized that downregulation of ERCC6 and ERCC8 may result in lower DNA repair capacity, thus elevating cancer susceptibility by allowing unrepaired DNA damage to remain, ultimately leading to carcinogenesis.

Genetically determined factors may lead to inter-individual variation in protein expression and function. Population-based epidemiologic studies have shown that *ERCC6* and *ERCC8* polymorphisms have a significant impact on the risk of some human malignancies, including gastric cancer [10, 11, 16, 28–30]. *In vitro* evidence indicated that *ERCC6* polymorphisms may alter protein function [16, 31], and reduced ERCC6 protein levels were associated with increased cancer risk [32, 33]. However, it is unclear whether the interactions of *ERCC6*-*ERCC8* SNPs influence protein expression in gastric mucosa, which could help to reveal hidden heritability for gastric carcinogenesis. Therefore, we further investigated the functional relevance of *ERCC6* and *ERCC8* SNPs on gene expression in a fixed group (CSG) at protein level. We found that single SNP had no correlation with corresponding gene expression. As for joint analysis of two SNPs, the *ERCC6* rs1917799–*ERCC8* rs158572 pair had significant effect on ERCC6 and ERCC6-ERCC8 expression. The lowest protein expression was observed in TT–GA/GG combined genotype, and GT/GG–GA/GG genotype had the highest protein expression. Interestingly, the TT–GA/GG combination was found to have a correlation with increased GC risk, and GT/GG–GA/GG genotype can decrease CAG risk. *ERCC6*_rs1917799 polymorphism is located in 5′ regulatory region of *ERCC6* gene and predicted having promoter activity. *ERCC8*_ rs158572 is located in region of intron in *ERCC8* gene and predicted having enhancer activity (FASTSNP, http://fastsnp.ibms.sinica.edu.tw/pages/input_CandidateGeneSearch.jsp). It is reported that distal regulatory enhancer elements in the genome can interact with proximal promoter regions to regulate the target gene's expression, and variants that change such interactions will cause the target gene to be dysregulated [34, 35]. We speculated that GT/GG-GA/GG genotype combination may activate such enhancer-promoter interaction to upregulate the gene expression, whereas there is no such effect for TT-GA/GG genotype. The regulation of gene expression by SNP combination may partially explain why individuals with specific genotypes had decreased risk of CAG or increased risk of GC.

In summary, *ERCC6* rs1917799, *ERCC8* rs158572 and rs158916 demonstrated pairwise epistatic interactions to affect CAG and GC risk. Expression of ERCC6, ERCC8 and ERCC6-ERCC8 combination have similarities that higher positivity was observed in CSG than CAG and GC. The *ERCC6* rs1917799–*ERCC8* rs158572 pair had significant influence on ERCC6 and ERCC6-ERCC8 expression. The regulation of SNP combination on gene expression may provide the genetic evidence for phenotypic variations of gastric diseases. Nevertheless, more thorough functional analysis will verify the interactions of genetic variation and gene expression between *ERCC6* and *ERCC8* in gastric carcinogenesis.

## MATERIALS AND METHODS

### Study population

The design of this study was approved by the Human Ethics Committee of the First Affiliated Hospital of China Medical University (Shenyang, China) before the outset of the research. Written informed consent was obtained from each study participant.

For the genetic association study, 1916 subjects were recruited from a population-based, combined serologic/endoscopic screening program for GC in the Zhuanghe area of Liaoning Province between 1997 and 2011. The screening population selection and recruitment process were reported previously [36]. All the enrolled subjects were diagnosed based on the gastroscopic and histopathological examinations by two independent pathologists. Patients with a history of other malignant tumors were excluded from our study. Histopathological findings were assessed according to the visual analog scale of the updated Sydney System for gastritis [37] and the World Health Organization (WHO) criteria for GC [38]. A 5-ml fasting venous blood sample was obtained for DNA isolation. The segregated blood clots were immediately frozen and stored until analysis. All the enrolled subjects were histologically classified into three groups: GC, CAG, and CSG (healthy control subjects with relative normal mucosa or only mild superficial gastritis). The demographic and geographic characteristics of study participants are shown in Table 6.

**Table 6:** Baseline characteristics of the subjects for the genetic association and the protein expression study

Variable	N	Subjects			*P*
		GC	CAG	CSG	
**The genetic association study**					
**Total n**	1916	n=467	n=700	n=749	
**Age**					<0.001
<60	1292	263	487	542	
≥60	624	204	213	207	
**Gender**					<0.001
Male	1109	321	401	387	
Female	807	146	299	362	
***H. pylori*-IgG**					<0.001
Positive	830	167	420	243	
Negative	1086	582	280	224	
**The protein expression study**					
**Total n**	327	109	109	109	
**Age (years)**					0.364
<60	186	59	59	68	
≥60	141	50	50	41	
**Gender**					0.84
Male	212	73	70	69	
Female	115	36	39	40	

CSG: chronic superficial gastritis; CAG: chronic atrophic gastritis; GC: gastric cancer.

For the protein expression study, tissue samples were obtained from 109 GC patients from the anorectal department of the First Affiliated Hospital of China Medical University, who underwent surgical treatment, without neoadjuvant chemoradiotherapy or other therapy between 2012 and 2015. Additionally, 109 individuals with CSG and 109 patients with CAG were randomly matched from the above mentioned genetic association study. There was no statistical difference among the groups in terms of age and gender composition (Table 6).

### SNP genotyping

Whole blood from individuals was collected, and blood clots were allowed to form by incubating clot-activating tubes at room temperature for 1 h. Each clot was transferred to a 2-ml centrifuge tube and stored at −80°C until DNA extraction. Genomic DNA of the blood samples from included subjects was extracted from venous blood as previously described using routine phenol–chloroform method [39]. For genotyping, the DNA concentration was adjusted to 50 ng/μl. SNP genotyping was performed using Matrix assisted laser desorption/ionization time of flight mass spectrometry, as previously described [40]. Briefly, all samples were placed randomly into 384-well plates and blinded for disease status. The genotyping assay was performed by CapitalBio (Beijing, China) using the Sequenom MassARRAY platform (Sequenom, San Diego, CA, USA). To evaluate the quality of the genotyping, 5% samples were repeatedly genotyped and the consistency rate was higher than 99%.

### *In situ* protein expression by immunohistochemistry

Paraffin-embedded tissues were sectioned into 4-μm-thick sections, and mounted onto positive-charged glass slides. Briefly, slides were deparaffinized in xylene, rehydrated in a graded alcohol series, washed in tap water and boiled at 95°C for 30 min in citric acid buffer (pH 6.0) for antigen retrieval. Endogenous peroxidase activity was blocked by washing with 3% hydrogen peroxide solution for 10 min, and the sections were then washed with phosphate-buffered saline (PBS, pH 7.4). Tissue collagen was blocked to avoid nonspecific binding by adding 10% normal goat serum at 37°C for 30 min. The sections were incubated with primary antibodies against ERCC6 (TA313375, 1:300, Origene, Rockville, MD, USA) and ERCC8 (AV31542, 1:500, Sigma, Saint Louis, MO, USA) at 37°C for 1 h. After rinsing three times with PBS, the sections were incubated with biotinylated secondary goat anti-rabbit antibody (Maixin, Fuzhou, Fujian, China), and then with streptavidin-biotin peroxidase for 10 min each. After secondary antibody staining, diaminobenzidine (DAB) was used as the chromogen for 1 min. Finally, slides were rinsed with water, counterstained with hematoxylin blued in water, dehydrated, cleared in xylene, and mounted.

### Standards for immunohistochemistry evaluation

ERCC6 and ERCC8 staining were mainly located in the cell membrane and cytoplasm. Staining results were evaluated independently by two pathologists blinded to the clinicopathological characteristics of patients. ERCC6 and ERCC8 expression were scored using a semi-quantitative method that took into account both the staining intensity and the percentage of cells at that intensity [41]. For each sample, staining intensity was scored according to intensity (0, no staining; 1, mild staining; 2, moderate staining; 3, strong staining) and range (0, ≤5%; 1, 5%–25%; 2, 25%–50%; 3, 50%–75%; 4, ≥75%) of the staining. Finally, the staining intensity and the staining range were multiplied to generate an immunoreactivity score (IS) for each sample, which was divided as: no staining, 0; mild staining, 1–4; moderate staining, 5–8; severe staining, 9–12. A score of 0 meant negative expression, while all others indicated ranges of positive expression.

### Detection of the serum *H. pylori* IgG titer

We tested the serum *H. pylori* immunoglobulin G (IgG) antibody titer by enzyme-linked immunosorbent assay (*Helicobacter pylori* IgG kit; Biohit, Helsinki, Finland) according to a previously described method [42]. Patients with a serum titer > 34 IU were diagnosed as *H. pylori* positive.

### Statistical analysis

This study defined the heterozygote plus rare homozygote compared with the wild-type genotype as the dominant model, the mutant genotype compared with the wild-type genotype plus the heterozygote as the recessive model [14]. The distribution of demographic characteristics and the genotypes in case and control groups was tested using chi-square test. Multivariate logistic regression analysis adjusted by age and sex was used to assess the OR and 95% confidence interval for the GC and CAG risks. The log likelihood ratio test was used for the interaction analysis between SNPs. Chi-square test was used to analyze differential expression of ERCC6 and ERCC8 between different gastric diseases and the correlation between SNP and gene expression. All statistical tests were performed using SPSS 18.0 (SPSS Inc., Chicago, IL, USA). *P* values less than 0.05 were considered statistically significant.

## SUPPLEMENTARY TABLES



## Abbreviations

ERCC6: Excision repair cross-complementing group 6
ERCC8: Excision repair cross-complementing group 8
CSG: Chronic superficial gastritis
CAG: Chronic atrophic gastriitis
GC: Gastric Cancer
SNP: Single nucleotide polymorphism
NER: Nucleotide excision repair
TCR: Transcription-coupled nucleotide excision repair
CS: Cockayne syndrome
