# Supplementary material for: Epistatic SNP interaction of ERCC6 with ERCC8 and their joint protein expression contribute to gastric cancer/atrophic gastritis risk
Source: Oncotarget. 2017 May 11;8(26):43140–52. doi: 10.18632/oncotarget.17814 (PMC5522134; doi:10.18632/oncotarget.17814)
Supplement: Supplementary file 1 [file oncotarget-08-43140-s001.pdf]

Epistatic SNP interaction of *ERCC6* with *ERCC8* and their joint protein expression contribute to gastric cancer/atrophic gastritis risk

SUPPLEMENTARY MATERIALS

SUPPLEMENTARY TABLES

Supplementary Table 1: Effects of a single SNP on ERCC6 and ERCC8 expression

SNP genotypes		Positive (%)	*P* value
***ERCC6* SNP and protein expression**			
**rs1917799**	**TT**	44(83.0)	ref.
	**GT**	41(95.3)	0.059
	**GG**	12(92.3)	0.403
	**GT/GG:TT**	53(94.6)	0.053
	**GG:GT/TT**	85(88.5)	0.684
***ERCC8* SNP and protein expression**			
**rs158572**	**AA**	82(91.1)	ref.
	**GA**	16(88.9)	0.767
	**GG**	1(100.0)	0.755
	**GA/GG:AA**	17(89.5)	0.822
	**GG:GA/AA**	98(90.7)	0.750
**rs158916**	**TT**	75(91.5)	ref.
	**CT**	21(87.5)	0.559
	**CC**	3(100.0)	0.597
	**CT/CC:TT**	24(88.9)	0.688
	**CC:CT/TT**	96(90.0)	0.577

Supplementary Table 2: The three dimensions interactions of the *ERCC6* SNP-*ERCC8* SNP- *H. pylori* infection status with the risk of GC/CAG

SNP genotypes			CAG vs. CSG		GC vs. CSG	
			P	**OR(95%CI)**	***P***	**OR(95%CI)**
ERCC6* rs1917799-*ERCC8* rs158572-*H. pylori						
TT	AA	(−)		1(ref)		1(ref)
TT	AA	(+)	<0.001	5.32(3.51-8.05)	<0.001	4.99(3.01-8.08)
TT	AG+GG	(−)	0.319	1.41(0.72-2.74)	0.046	2.16(1.01-4.61)
TT	AG+GG	(+)	<0.001	7.74(2.80-21.4)	<0.001	12.5(3.99-39.2)
GTGG	AA	(−)	0.507	0.90(0.65-1.23)	0.090	1.39(0.95-2.04)
GTGG	AA	(+)	<0.001	5.14(3.62-7.30)	<0.001	5.49(3.58-8.40)
GTGG	AG+GG	(−)	0.058	0.58(0.33-1.02)	0.147	1.54(0.86-2.74)
GTGG	AG+GG	(+)	0.001	2.74(1.52-4.94)	<0.001	3.83(1.96-7.47)
			$P_{interaction}$=0.771		$P_{interaction}$=0.466	
			interaction index=0.80		interaction index=0.55	
ERCC6* rs1917799-*ERCC8* rs158916-*H. pylori						
TT	TT	(−)		1(ref)		1(ref)
TT	TT	(+)	<0.001	5.52(3.53-8.64)	<0.001	5.40(3.12-9.35)
TT	CT+CC	(−)	0.812	1.07(0.62-1.86)	0.266	1.44(0.86-2.72)
TT	CT+CC	(+)	<0.001	4.94(2.51-9.72)	<0.001	5.62(2.58-12.2)
GTGG	TT	(−)	0.491	0.89(0.64-1.24)	0.041	1.52(1.02-2.28)
GTGG	TT	(+)	<0.001	4.66(3.23-6.71)	<0.001	5.39(3.45-8.41)
GTGG	CT+CC	(−)	0.038	0.59(0.36-0.97)	0.909	0.97(0.56-1.68)
GTGG	CT+CC	(+)	<0.001	4.01(2.36-6.79)	<0.001	3.82(2.01-7.25)
			$P_{interaction}$=0.459		$P_{interaction}$=0.517	
			interaction index=1.54		interaction index=1.54	

CSG: chronic superficial gastritis; CAG: chronic atrophic gastritis; GC: gastric cancer.
